# A Wireless Sensor Network-Based Approach with Decision Support for Monitoring Lake Water Quality

**DOI:** 10.3390/s151129273

**Published:** 2015-11-19

**Authors:** Xiaoci Huang, Jianjun Yi, Shaoli Chen, Xiaomin Zhu

**Affiliations:** 1 Department of Mechanical Engineering, East China University of Science and Technology, Shanghai 200237, China; E-Mails: hxc8011@163.com (X.H.); pariscat@aliyun.com (S.C.); zxmin4236@163.com (X.Z.); 2 Institute of Automotive Engineering, Shanghai University of Engineering Science, Shanghai 201620, China

**Keywords:** WSN, decision, monitor, lake water

## Abstract

Online monitoring and water quality analysis of lakes are urgently needed. A feasible and effective approach is to use a Wireless Sensor Network (WSN). Lake water environments, like other real world environments, present many changing and unpredictable situations. To ensure flexibility in such an environment, the WSN node has to be prepared to deal with varying situations. This paper presents a WSN self-configuration approach for lake water quality monitoring. The approach is based on the integration of a semantic framework, where a reasoner can make decisions on the configuration of WSN services. We present a WSN ontology and the relevant water quality monitoring context information, which considers its suitability in a pervasive computing environment. We also propose a rule-based reasoning engine that is used to conduct decision support through reasoning techniques and context-awareness. To evaluate the approach, we conduct usability experiments and performance benchmarks.

## 1. Introduction

When monitoring lake water quality, one must deal with a very complex and harsh environment. Wireless sensors are used to meet the demands of real-time online monitoring over large water areas, complex terrains, or remote areas. WSN have been extensively used for environmental monitoring, forest fire prevention, and military applications. A WSN is a self-organized wireless network composed of a large number of sensors. WSN nodes typically use an independent power supply, which means they can be easily deployed in large-scale, complex environments. However, this also means that WSN nodes have very limited energy, memory, and computing resources. It is widely recognized that we must determine effective monitoring and management methods for WSN nodes. Consider the example of lake water quality monitoring using a WSN. This can be a harsh or dangerous environment. In this case, it is typically difficult to change energy sources. Even if we can replace the node’s energy supply, the process is expensive. For lake water quality monitoring WSNs, instead of using alternative energy sources, we typically implement effective strategies for reducing the energy consumption and prolonging the life of the network. Suitable management methods for WSNs significantly improve the network performance, reduce energy consumption, and prolong the lifetime of the entire network.

In this paper, we propose an OWL ontology and SWRL-based knowledge description and semantic reasoning method. This technique is aimed at state monitoring and decision support for WSNs applied to lake water quality monitoring. Our proposed ontology is based on state changes and is an extension of the SSN ontology of SSN-XG and the WSN ontology proposed by Bendadouche *et al.* [1,2]. In our method, the WSN state and corresponding monitoring methods are encoded in the ontology, which is then used to manage the WSN and for self-configuration of the network nodes. Although we do not use the exact same structure as the existing WSN ontology, it does have good compatibility and scalability so that it can be applied in pervasive computing environments. The ontology of the water quality monitoring system manages the WSN according to the nodes’ energy, data, networking, observation, and water quality states. To reduce the energy consumption and prolong the life of the network, we have developed a rule set that contains monitoring and management rules developed using SWRL. Managers can select rules according to different monitoring targets. The rule set can be edited and extended, so that different logical rules can be incorporated using a common language. In this paper, we combine the rule set with the ontology to help WSN make intelligent decisions for self-configuration.

The remainder of this paper is organized as follows: the next section provides an overview of related WSN management systems. In Section 3.1, WSN challenges in a lake water quality monitoring environment are discussed. In Section 3.2, we propose an ontology based on state monitoring and in Section 3.3 we present our SWRL-based reasoning rules for the self-configuration of WSN nodes. Then Section 3.4 describes the workflow of the proposed approach in the system. In Section 4, we describe the evaluation and benchmark experiments, and Section 5 contains our conclusions and directions for future work.

## 2. Related Work

Several studies have focused on the management of WSNs, which are important components of complex environmental monitoring systems. A large number of techniques have been applied to manage specific WSNs. For example, an energy-efficient method was proposed in [3] for an ant-based routing algorithm in Wireless Sensor Networks. In [4], simple and feasible synchronous node sleeping and waking mechanisms for small-scale WSNs were proposed. Sensor nodes were divided into forwarding nodes and listening nodes. A beacon frame containing a sleep command sent from the coordinator can be sent to the listening nodes via the forwarding nodes. In [5], an Energy-Efficient Adaptive Geosource Multicast Routing (EAGER) was proposed for WSNs. It addresses the energy and scalability issues of previous location-based stateless multicast protocols in WSN. A complete hierarchical key management scheme that uses symmetric cryptographic algorithms and low-cost operations for heterogeneous cluster-based WSN was proposed in [6]. The scheme consists of four types of keys for each sensor node: an individual key, a cluster key, a master key, and pairwise keys. However, it is very complicated and time consuming to incorporate and manage different sensor networks. Additionally, we must use different techniques to manage different networks. Most sensor network management models use their own scenarios and management languages. These languages may be quite different, because each network has its own language. Therefore, we require a method that can be applied to a pervasive computing environment.

Semantic web technologies have recently been proposed to enable interoperability of sensors and sensing systems. An ontology can be combined with semantic web technology standards to form a semantic sensor web. An ontology is a conceptual model of abstract concepts related to some phenomena in the objective world. The description represented by an ontology is independent of specific environmental conditions. Ontology concepts and their constraints are clearly defined. An ontology is readable and can be processed by a computer. It is based on groups rather than individuals, expresses common knowledge, and reflects a concept set of related areas. We can combine a WSN environment and an ontology to facilitate knowledge sharing, conceptualize the WSN’s knowledge, and derive a commonly recognized language. By constructing a WSN ontology, we can integrate different WSN management technologies and apply them to pervasive environments.

The Sensor Web Enablement (SWE) [7] initiative of the Open Geospatial Consortium (OGC) defined data encodings and web services for storing and accessing sensor-related data. These standards provide syntactic interoperability. Examples include SensorML and O&M [8,9,10]. Some scholars have proposed effective Semantic Sensor Network (SSN) ontologies based on this research. The W3C Semantic Sensor Network Incubator Group (SSN-XG) [1,11] produced OWL (Web Ontology Language) to describe sensors and observations, *i.e.*, a SSN ontology. This SSN ontology can describe sensors in terms of their capabilities, measurement processes, observations, and deployments [1,11]. The SSN ontology was extended to WSN by Bendadouche *et al.*, and was used to optimize the life of a network [2]. It deduces the state of the node based on its energy level. However, the authors did not provide details of the reasoning methods. To effectively manage a WSN, we should consider the objectives and environmental parameters using certain logical rules. Although these ontologies are compatible, specific applications require supplemental rule descriptions and reasoning methods.

Semantic Web Rule Language (SWRL) [12] is an expressive Web Ontology Language (OWL)-based rule language [13,14]. SWRL allows users to write rules that can be expressed in terms of OWL concepts to provide more powerful deductive reasoning capabilities than OWL alone. Its purpose is to drive Horn-like [12] rules in conjunction with the OWL knowledge base, which supply rule descriptions and reasoning. In [15], Cuppens-Boulahia *et al.* presented an approach for reacting to network attacks using an ontology to store policy information and to generate new security models. They used OWL and SWRL, and have the advantage of using existing generic tools for parsing and reasoning. OWL can be used to merge distributed ontologies. A semantic, state machine-based diagnosis approach for a web service-based middleware was proposed in [16]. They used OWL ontologies and SWRL to develop diagnosis and monitoring rules, which were based on state changes and invocation relationships. Malfunction information and resolution methods are encoded in an OWL ontology as part of the “device” ontology, and can be used at run time to resolve malfunctions and fulfill self-healing activities. SWRL rules at device and system levels were designed and executed as needed.

## 3. Materials and Methods

### 3.1. WSN Challenges in a Lake-Water Quality Monitoring Environment

**Figure 1:**
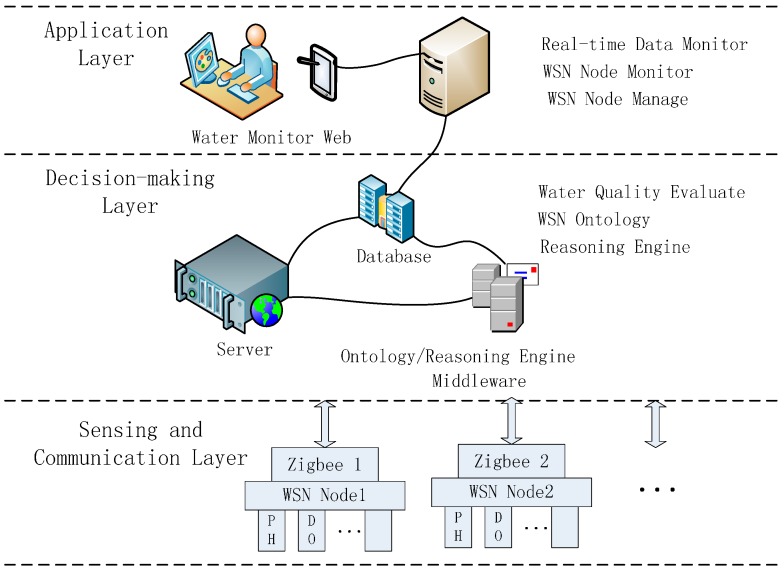
Structure of water-quality monitoring system.

In the lake water quality monitoring project, a number of monitoring nodes are distributed throughout the water and are connected to a sufficient number of water quality sensors. The data are aggregated to the gateway node through a ZigBee [17] WSN, and are sent to the remote center server by GPRS (general packet radio service) modules that act as gateway nodes. Any further data processing is conducted by software on the server. The architecture of the water quality monitoring system is shown in Figure 1. The system is composed of three layers: (1) sensing and communication; (2) decision making; and (3) application. Each layer is divided into components with different functions.

The sensing and communication layer is composed of monitoring nodes and gateway nodes. The monitoring area collects the water quality data, and contains a sufficient quantity of monitoring nodes. The decision-making layer mainly stores, analyzes, and manages the water quality data. It includes a water quality database in the remote monitoring center, and a model for assessing water quality. A user can remotely log in to this layer. The application layer mainly records interactions between the monitoring system and users. A user can check various types of data using the website.

Because the monitoring task is performed in a lake area, which is usually a harsh or dangerous environment, reconfiguring the WSN manually is difficult. Therefore, it is a challenge that the WSN should have the flexibility of self-configuration with smart decision support for special purposes. For example, to save energy, sensor nodes could take advantage of long periods of idle time between interesting events. For this reason, the requirements of WSN in lake water quality monitoring enumerated from the point of view of smart decision support are as follows:

(1) Flexibility in monitoring requirement. Given the varied requirements of the users, WSN configuration should be adapted to their target scenario. The flexibility of interaction between WSN and users is needed.
(2) Flexibility in task scheduling and execution. Many changing and unpredictable situations are observed in lake water environments. For example, some monitoring indicators (such as the pH level of water) may be particularly abnormal in a certain area or over a certain period and should be given close attention. The WSN should have flexibility in task scheduling and execution, and it should be able to re-plan how to proceed from this abnormal state.
(3) Flexibility in resource management [18]. WSN nodes have extremely limited energy, memory, and computing resources. Thus, the use of resources should not be static and predefined. The WSN should be able to change its resource configuration depending on the contextual information. For example, the WSN can switch the sample frequency and communication frequency to very slow if the battery has minimal power.
(4) Flexibility in data management. Large amounts of data are generated during water monitoring, but not all of these data are usable. The WSN can change some data sampling behaviors flexibly to make them suitable for different situations. Some irrelevant data are filtered to save data management time.

The focus of those requirements is to explore the capabilities of semantic rules in the area of self-configuration. The WSN, depending on the information extracted from its context, can decide autonomously on the configuration of its resources and services. The present study regards the reasoning engine as a middleware decision layer within the decision-making layer. The proposed middleware structure is service oriented. The decision-making layer provides services for the upper layer. The design has different functions to make it more flexible, so that it can easily adapt to unexpected changes. The proposed middleware based on the ontology includes diagnosis management and decision reasoning functions. The diagnosis management function diagnoses changes to the data state in the sensing and communication layer, or changes in the observation state in the application layer. For the realization of the decision reasoning function, with proposed WSN-based lake-water monitoring ontology, additional rules based on SWRL have been developed to extend the information inferred by the semantic framework.

### 3.2. Proposed WSN-Based Lake-Water Monitoring Ontology

#### 3.2.1. Basic Ontological Framework

**Figure 2:**
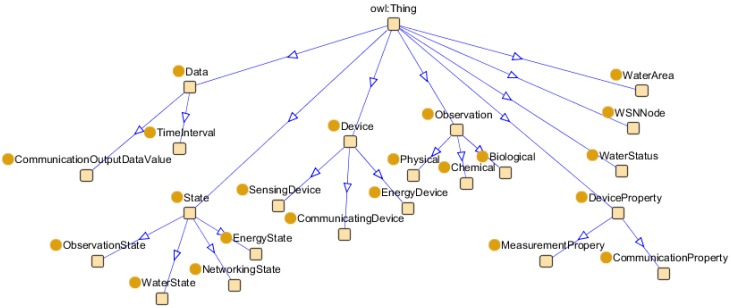
Top-level view of WSN ontology.

Our ontology builds on the ontology proposed by W3C SSN-XG [1] and Bendadouche *et al.* [2]. Our framework is versatile and scalable, and it aims to improve compatibility. The present study mainly focuses on methods for monitoring and managing WSN nodes from the energy consumption perspective. Considering a specific application, we did not need all the concepts and attributes in the SSN ontology. We used only the “sensor property” and “observation” classes, as well as their related properties. When describing the WSN concept, we focused on the description of the state machine. We used an ontology development tool called Protégé [19] to construct and edit our ontology, and we also used the OWL DL knowledge representation language [13]. We calculated the automatic hierarchical classification using RacerPro [20] and verified the consistency of the ontology logic. The top-level view of the ontology presented in Figure 2 describes the following:

(1) the WSN devices (for sensing, energy storage, and communication) and the device properties (measurement and communication);
(2) monitoring task of WSN (chemical, physical, and biological observation);
(3) environment status (water status, water area);
(4) WSN states (observation, energy, water, and networking states).

To deal with varying situations, depending on the states of WSN and environment context information, the proposed approach should self-configure WSN to perform certain tasks.

For example, the power source supplies the energy needed by the device to perform the task. Experimental measurements have shown that generally, data transmission is highly expensive in terms of energy consumption [21]. In other cases, the energy expenditure for data sensing may be comparable to, or even greater than, the energy needed for data transmission. The battery power in the energy device is limited although much of it is rechargeable [22]. Therefore, devices (such as those for communication or sensing) in the WSN should have self-configuration ability according to the energy quantity of the energy device. For example, the communicating frequency and sensing frequency should be self-alternated or should enter sleep mode. For this reason, a device state perspective is considered in the semantic knowledge description.

For another thing, large amounts of data are collected by sensing device and being processed and transmitted. Although several competitive and efficient routing algorithms for WSNs have been developed and surveyed for energy-saving purposes [22,23], excessive data are still generated, which are mostly redundant for water-quality monitoring. Therefore, a data state perspective is considered to minimize the data volume.

Sometimes users may have a specific monitoring scheme for different water areas or periods. Moreover, the water quality changes in different periods. Correspondingly, the WSN should have self-reconfiguration ability in accordance with observation demand and water quality for the purpose of saving energy. Therefore, an observation-demand state perspective and a water-quality state perspective are considered in our approach. To achieve the aforementioned level of reasoning, the semantic knowledge description is based on the following:

- a device state perspective,
- a data state perspective,
- an observation-demand state perspective,
- a water-quality state perspective.

#### 3.2.2. Semantic Knowledge in Ontology from the Device State Perspective

A WSN node typically includes sensing, communication, energy, and calculation modules. The sensing model contains sensors and other sensing devices, and records parameters such as temperature. The communication module communicates with other nodes, for example, by exchanging network information and transmitting data. The energy module provides energy for all modules in the node.

**Figure 3:**
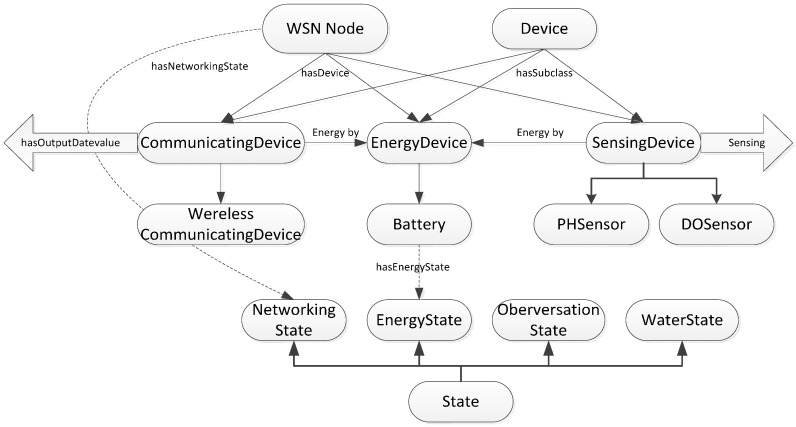
Description of sematic knowledge in ontology from device state perspective.

The device class contains the subclasses of perception, communication, and energy. They are “Communicating Device”, “Energy Device”, and “Sensing Device”, as shown in Figure 3.

The state of the device is diagnosed by the “Energy State” and “Networking State” subclasses, which means that a WSN device is typically associated with two kinds of state. An energy device such as a battery is linked to the “Energy State” individual through the “has Energy State” property. The “Energy State” instance is the percentage of remaining battery power. The “has Networking State” property links a WSN node to the “Networking State” instance. The “Networking State” instance shows if the networking state is either “Open” or “Closed”.

#### 3.2.3. Sematic Knowledge in the Ontology from the Data State Perspective

**Figure 4:**
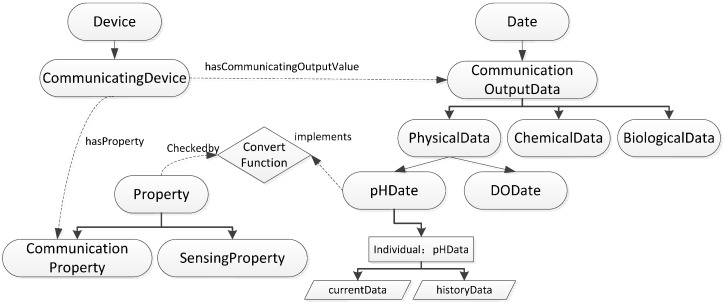
Sematic knowledge in ontology from data state perspective.

The communicating device’s transmitting data are sensed by the WSN node. The “has Communicating Output Value” property is linked to “Communication Output Data Value”, which is a subclass of the “Data” class. The communicating and sensing devices have communicating characteristics and perceptual characteristics; for example, the communicating frequency and voltage of the data transceiver, which are typically related to the node’s energy consumption. The device’s performance is judged according to changes to some output calibrated data; for example, an adjustment to the communicating frequency. So the “Communication Output Data Value” instance records the current and historical data as input parameters of the state function, as shown in Figure 4.

#### 3.2.4. Sematic Knowledge in Ontology from the Observation State Perspective

Parameters such as the temperature, humidity, wind speed, and other physical phenomena are observed by the system. The observed object is typically set by the user according to their requirements. Parameters such as the pH and dissolved oxygen (DO) are recorded when monitoring water quality. These physical phenomena cannot always be directly recorded by the sensor, and instead require indirect methods that are converted to output signals that are suitable for communication and measurement. For example, a thermistor measures changes to electrical resistance as a proxy for temperature. Many ontologies consider the pattern of stimulus-sensor-observation [1]. In this paper, we are only concerned with observed objects monitored by system, so we have not considered perception methods for a specific sensor. “Sensing Device” is directly linked to the physical object (observation) through the “has Observation” property.

**Figure 5:**
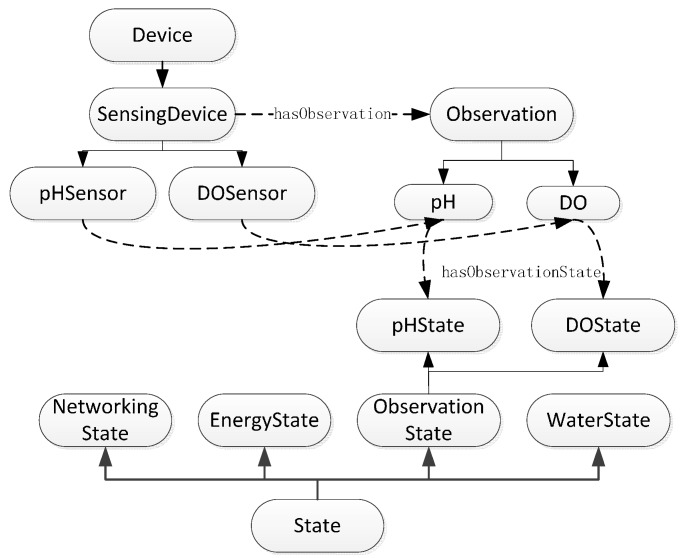
Sematic knowledge in ontology from observation state perspective.

“Observation State” is linked to the “Observation” individual by the “has Observation State” property, which is a subclass of the “state” class. The observation state refers to whether a physical object needs to be observed. Sometimes only parts of the observations must be monitored by a WSN, which are set by the “observation state”. The relationship between the observed object and the observation state is shown in Figure 5.

#### 3.2.5. Sematic Knowledge in the Ontology from Water Quality State Perspective

In this application, we are mainly concerned with the water quality status. We can adjust the energy consumption of the WSN according to the water quality state. For example, when the water quality is low or some observation index exceeds the standard range, we can increase the sampling and communication frequencies. Then, the energy consumption will increase. However, if the water quality grade is high, then we can use lower frequencies or a sleeping state to save energy.

**Figure 6:**
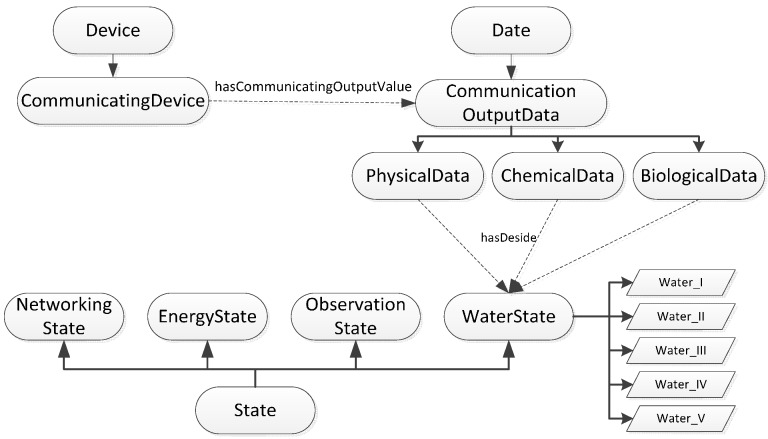
Sematic knowledge in ontology from water quality state perspective.

As shown in Figure 6, “Water State” is a subclass of the “state” class and has five levels: “Water_I”, “Water_II”, “Water_III”, “Water_IV”, and “Water_V”. The water quality state is determined using a test, so “Communication Output Data” has the “hasDeside” property, which links it to the “Water State” individual.

### 3.3. Reasoning Engine and Rules Based on SWRL

Dynamically controlling the WSN configuration is a complex task that requires intelligent inference based on specific working state and requirements. Owing to this requirement, with ontology used in memory, additional rules have been developed to extend the information inferred by the semantic framework.

As an alliance to OWL, SWRL can be used to write rules to reason about OWL individuals and to infer new knowledge about those individuals. SWRL is an expressive OWL-based rule language, which can be used to write rules to reason about OWL individuals and to infer new knowledge about those individuals. A SWRL rule contains an antecedent part, which is referred to as the body, and a consequent part, which is referred to as the head. The body and head consist of positive conjunctions of atoms. Informally, a SWRL rule may be read as meaning that if all the atoms in the antecedent are true, then the consequent must also be true. In SWRL rules, the symbol “^” represents conjunction, “?x” represents a variable, and “!” represents implication. If there is no “?” in the variable, then it is an individual. A SWRL editor is available as part of Protégé-OWL [24].

Using the previously proposed ontology, our objective was to dynamically control a WSN’s energy consumption and design a set of SWRL reasoning rules. The complexity of dynamic control methods for WSN energy consumption means that a variety of control strategies have been proposed. In this paper, we only considered controlling the sample and communication frequencies, by dynamically switching the WSN between low frequencies, high frequencies, or sleep to save energy. Our rules are extendable, which means that we can conveniently add more complex or more reasonable inference rules.

#### 3.3.1. Energy Rules

An energy rule is used to control the communication frequency according to the energy state of the batteries. When the battery energy is more than 50%, the communication device uses higher frequencies; when it in the range of 10%–50%, the communication device uses lower frequencies; and when the battery energy is lower than 10%, the communication device and sensor enter a dormancy state to avoid energy depletion. Sampling and communicating intervals of 0 represent the dormant state. In order to do this, the rule defines a situation using the stored knowledge (e.g., “there is a WSN node, this node has a battery, a communication device and some measurement device. The battery has very-low energy, and the communicating frequency or the measurement frequency is not slow”), and defines an action for it. This action consists in dropping a particular triple (the frequency of the communication device and the measurement device of WSN) and replacing it by a new one.

**Figure 7:**
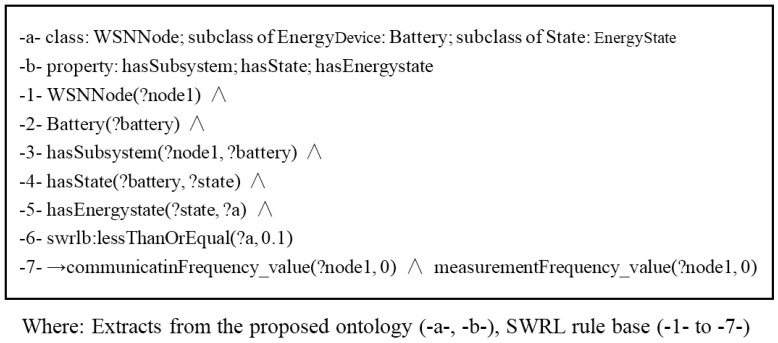
Example of self-configuration with energy rules when battery energy is low.

An example of self-configuration with energy rules when battery energy is low is presented in Figure 7. From the proposed ontology definitions (see above extracts), some classes (-a-) are extracted for inference. The extracted property (-b-) is their initial relations. From (-1-) to (-7-) is the inference procedure according SWRL rule. First, some individuals are identified to be member of the extracted classes. The node1 individual is identified to be “WSN Node” since it is a WSN node. The battery (-2-) is identified to be Battery which is a subclass of “Device” class and is a subsystem of “WSN Node” class (-3-). Individual “a” is identified to be “Energystate” which is a state of “battery”(-4-, -5-). Second, from these initial facts, executing the rule engine with the SWRL rule base, and then finally enables to infer the communication device and sensor enter a dormancy state (-6-, -7-). This example also show how SWRL rules are mapped to ontology.

#### 3.3.2. Data Rules

**Figure 8:**
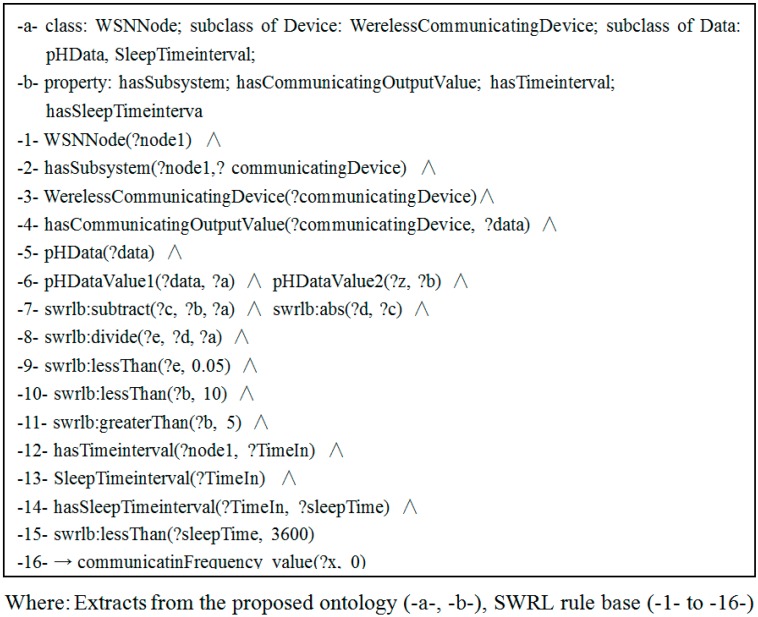
Example of self-configuration with data rules when the data value does not exceed a specific limit.

A data rule is used to monitor changes to the output data of a certain observed object. Such as the one shown in Figure 8, when the battery energy is lower than 5%, the data value does not exceed a specific limit, and the node sleep time is not more than 1 h, the communication device enters a dormant state.

#### 3.3.3. Networking State Rules

**Figure 9:**
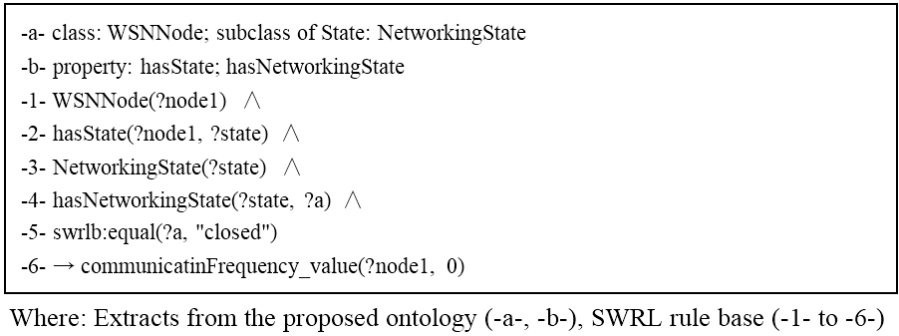
Example of self-configuration with networking state rules when disconnected from the network.

When disconnected from the network, a WSN node samples the data but does not communicate. Such as the example shown in Figure 9, there is an error of disconnection from the network taking place in WSN, then the WSN establishing a task of making the communication device to enter a dormancy state.

#### 3.3.4. Observation Demand State Rules

**Figure 10:**
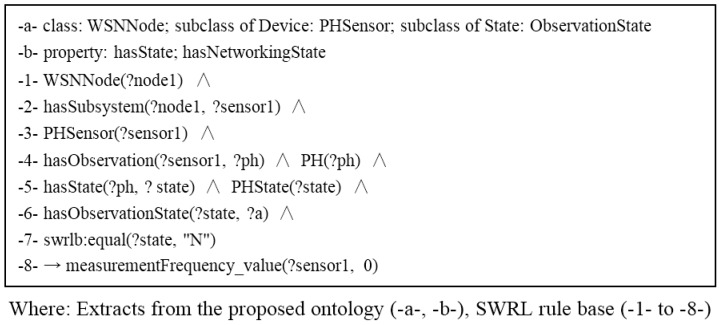
Example of self-configuration with observation-demand state rules.

To monitor water quality, a WSN node may have numerous sensors [25,26]. However, in a certain area or over a certain period, a user may not need to monitor the entire object. The energy needed for data sensing is substantial. To save energy, sensor nodes could take advantage of long periods of idle time between interesting events. During periods of inactivity, the sensors can gradually scale back their energy consumption [27]. Several approaches have been proposed to solve the problem of turning off the sensors [28,29]. Then, an observation can be set to an operational or dormant state according to specific requirements. The example in Figure 10 shows that a pH sensor enters a dormancy state when the pH is not observed by users.

These configurations are later defined by the WSN. The user can provide minor indicators (e.g., warning value of battery energy) so that the WSN can be adopted without needing to deal with the code by changing the rule files. The rules are abstract enough enabling sharing the same rules among different types of WSN.

### 3.4. Deployment of the Proposed Approach

**Figure 11:**
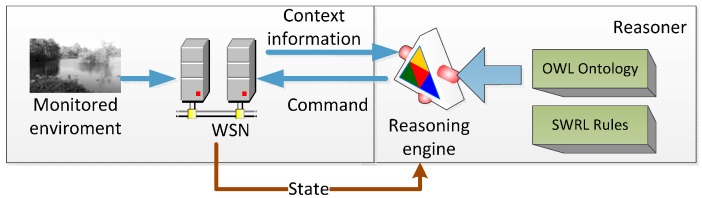
Work diagram of proposed approach.

The following steps show the work flow of the proposed approach during execution time. The process is shown in Figure 11. (1) Extracting relevant information from the context: the WSN extracts relevant information on itself and the monitored environment. The context information is directly mapped to the ontology; (2) Updating the instantiated ontology: the instantiated ontology is updated with new information. The values of the instantiated ontology change accordingly; (3) Rule selection and decision making: the reasoning engine recognizes the WSN states and finds which rules should be activated, as well as makes intelligent decisions; (4) Command execution: the WSN receives the command from the reasoning engine and acts consequently. The reasoning engine can be customized to the operating parameters of a WSN according to its particular application.

**Figure 12:**
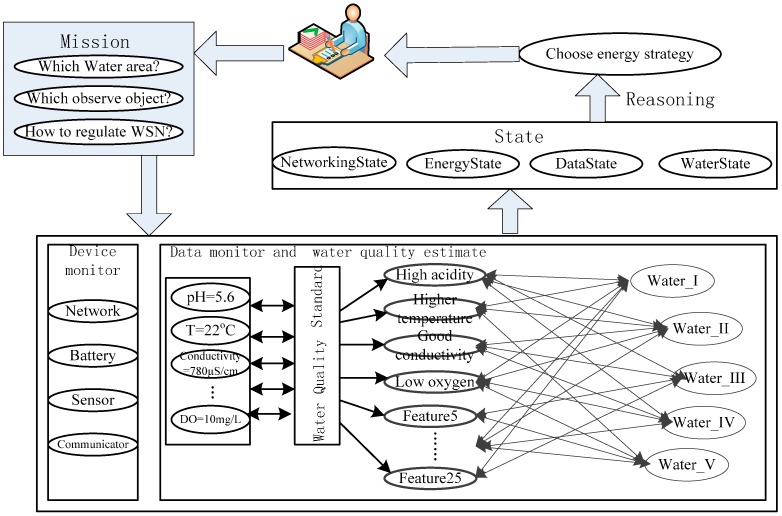
Workflow for case study.

With an example of decision-making on energy strategy, the entire process is shown in Figure 12. Monitoring tasks are set by a manager and conducted by the WSN. The monitoring middleware uses a database that is mainly responsible for receiving the data and some preprocessing. 

When the system is working, the database is connected through SQL and creates a communication thread for receiving and processing data. When processing is complete, data are stored in the corresponding database table using an SQL insert statement. The collected data are used to judge the water quality on the basis of an evaluation standard. The state machine monitors changes in the devices, data, observations, and water quality of the WSN. Any changes to these states are used to formulate an energy strategy. The decision-making steps based on the ontology and reasoning rules are as follows:

*Step 1: Instantiation of current ontology.* The basic ontological structure is shown in Figure 1, and the reasoning rules have been introduced in Section 3.2. However, this is a basic program and cannot be directly processed by the inference engine. In this step, we create instances according to the known conditions. This case is mainly concerned with controlling the energy according to changes in the observed data, the main instances are “WSN Node”: “WSN Node-1”, “Water Area”: “Water Area-1”, “Communicating Device”: “Wireless Communicating Device1”, “pH Data”: current Data, “pH Data”: history Data, “Communication Property”: “Communication Frequency”, and “Sensing Property”: “Sample Frequency”.

**Figure 13:**
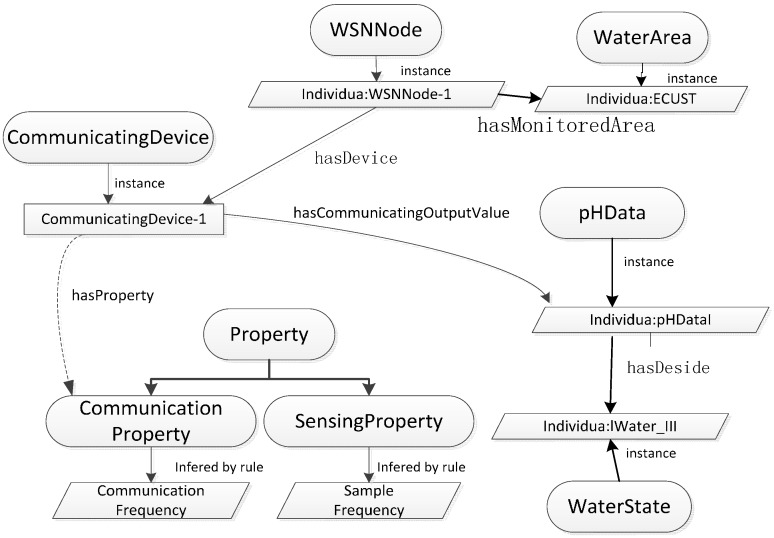
Some property contacts between instances.

*Step 2: Creating object property contacts between instances.* In the ontology, properties are defined for all classes. After the instances are defined, the property contacts should be appended to each instance in the Protégé editor, as shown in Figure 13.

**Figure 14:**
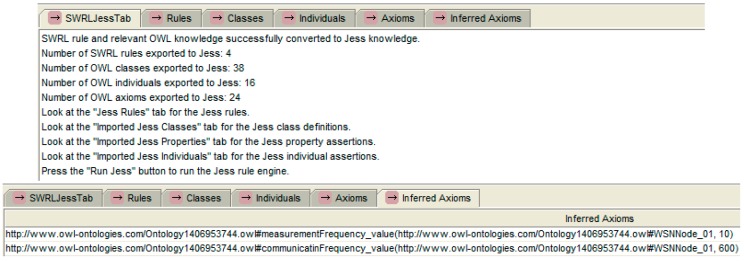
Decision result in Jess.

*Step 3: Reasoning according to SWRL rules.* This step requires a reasoning engine such as Jess [24]. Jess is a Java-based rule engine that consists of a rule base, fact base, and an execution engine. Jess combined with SWRL is a SWRLJessTab plug-in provided in the Protégé-based tools. The Jess inference engine runs in the Protégé editing environment, which displays the reasoning results. It uses the previously defined data rules. The decision result from Jess is presented in Figure 14.

*Step 4: Implementation of decision results.* The decision results are converted to OWL files. The application layer reads the analysis results and uses them to determine if it should reconfigure the WSN.

## 4. Evaluation and Experiments

### 4.1. Evaluation

**Figure 15:**
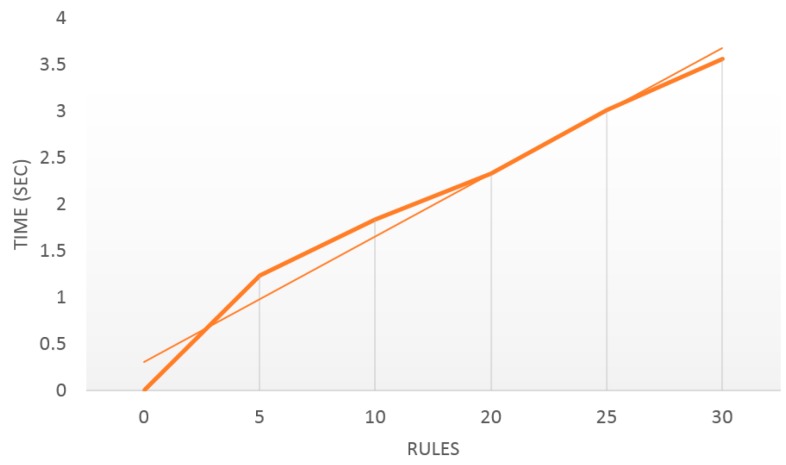
Number of rules (horizontal) and time for updating the ontology.

To check if the time required by the solution in this system is acceptable, the following software platform is used to measure performance: Protégé 3.4.4, JVM 1.6.02-b06, and Windows 8.1. The hardware platform is Core2Duo 2G HZ CPU and 2G DDR2 RAM. The size of the WSN ontology is 270,336 bytes and the ontology contains 30 rules. The effect of the number of rules on performance has been analyzed. Measurements have been taken and the trend is presented in Figure 15, which shows the real time used and the linear trend line. We can observe that the time taken has a linear relationship with the rules needed to be processed. The maximum value is 3.56 s. The results show that the total execution time is not very high and enough for the water-quality monitoring.

### 4.2. Experiments

The previous sections introduced the ontology framework and proposed some SWRL reasoning rules for controlling energy consumption. The functions and performance parameters of all systems should be verified to determine whether the proposed monitoring function achieves the expected result. Because it is difficult to test battery energy in lake water area, therefore we design a testing platform in a laboratory environment.

#### 4.2.1. Construction of Testing Platform

**Figure 16:**
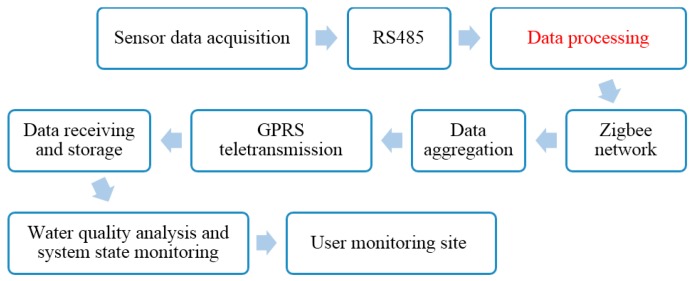
Online lake-water quality monitoring process.

The online lake water quality monitoring process is shown in Figure 16.

**Figure 17:**
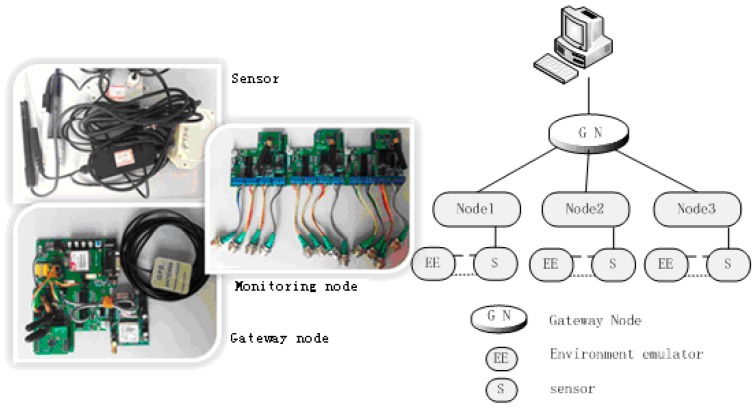
Testing platform for a lake water quality monitoring system.

We constructed a testing platform in a laboratory environment, debugged and tested the function of each module, and assessed the reliability of the system. Sensors, monitoring nodes, gateway nodes, and other components must be properly combined together. The test platform for this experiment is shown in Figure 17. It consists of three monitoring nodes, gateway nodes, sensors, and a PC.

The monitoring nodes are located in the bottom layer of the system. The monitoring nodes read and process sensor data, and communicate with the gateway node. A monitoring node contains power, data acquisition, wireless communication, and microprocessor control modules. It uses a storage battery as a power supply. The power module provides power to the other modules. The data acquisition module is a RS485 bus interface circuit and sensor module. It records the real-time water data, which is transmitted to the control chip for processing. The processor module is used to coordinate the operation of each module, to control the sensor data readings, and for receiving, processing, and transmission. The wireless communication module performs the ZigBee communication and networking functions. ZigBee technology is a kind of short distance, low complexity, low power consumption, low rate and low-cost two-way wireless communication technology [30]. The selected ZigBee communication module is developed by Mechatronics and the information laboratory of East China University of Science and Technology.

The gateway node controls Zigbee’s internal network, and joins the external and internal networks. It is the key point of the communication and data transmission process. The gateway node focuses on either the communication between the coordinator and each Zigbee node, or the communication between a GPRS module and the remote monitoring server. The gateway node is mainly composed of a power supply module, a microprocessor control module, an Electrically Erasable Programmable Read-Only Memory (EEPROM) module, a GPS information acquisition module, a Zigbee communication module, and a GPRS communication module. The microprocessor control module is used for data processing and coordinates the work of the other modules to ensure an orderly data communication process. The EEPROM memory module is used to store the data during processing, so that no data are lost. The GPS information acquisition module collects a real-time geographic position. The Zigbee communication module receives the data communicated by the monitoring nodes, and the GPRS communication module remotely transmits all kinds of received information to the monitoring center using a mobile network.

**Figure 18:**
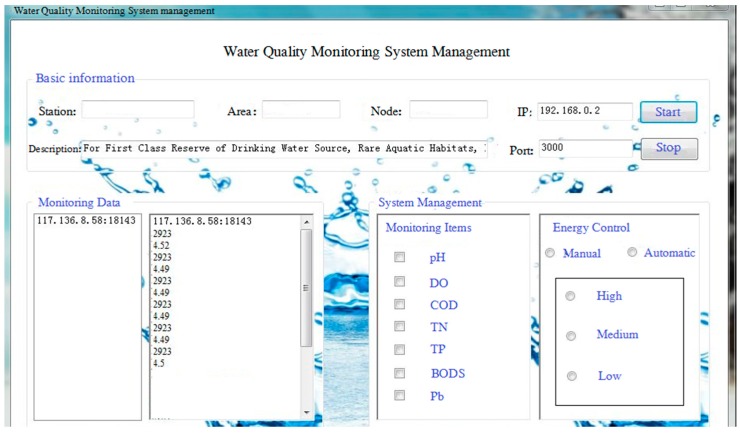
Monitoring system management interface.

In this system, real-time data and water quality analysis results for monitoring the lake were presented to users in the form of webpages. We also included a management function for an administrator. Figure 18 shows the visualization interface, which presents information about the received data and the system management. The design of the interface helps the administrator check the data and management options, and control the state of the system.

#### 4.2.2. Experimental Preparation

We use three monitoring nodes in our experiment. Each monitoring node has the same hardware composition as mentioned in the preceding sections. Each monitoring node communicates with the gateway node separately. In one monitoring node, two sensor nodes are connected. One sensor node has a pH sensor and water temperature sensor, and another has a dissolved oxygen (DO) sensor and conductivity sensor. To compute the energy consumption conveniently, the monitoring and gateway nodes are powered by two common 5 V, 800 mAh rechargeable batteries. The battery has an internal protection circuit to guard against overdischarge, undervoltage, and overcharge conditions. Five groups of water samples are adopted in our test. The water samples are from real water regions of Huangpi, Jiangmenkou, Gulao, Xihucun, and Rongjiang, which correspond to five grades of water quality in GB3838-2002. The definition of five grades of water quality in GB3838-2002 [31] is presented in Table 1.

**Table 1:** Definition of five grades of water quality in GB3838-2002.

Grade	Definition
Water_I	Mainly applicable to the source water or national nature preserves.
Water_II	Mainly applicable to the first level of centralized drinking water source protection area, rare aquatic habitat, fish spawning grounds, larvae feeding *etc.*
Water_III	Mainly applicable to the second level of centralized drinking water source protection area, fish and shrimp overwintering grounds, swimming channel, aquaculture areas.
Water_IV	Mainly applicable to general industrial water district and the recreational water area with non-direct contact with human body.
Water_V	Mainly applicable to agricultural water district and the general requirement of landscape waters

Some water quality data of five groups of water sample are shown in Table 2.

**Table 2:** Water quality data of five groups of water sample.

Water Quality Item	Group 1	Group 2	Group 3	Group 4	Group 5
Water temperature (°C)	24.5	24.5	24.5	24.5	24.5
pH	6.9	6.6	7.1	7.4	7.6
Conductivity (us/cm)	276	430	657	1543	2000
DO (mg/L)	7.3	5.9	4.7	2.85	1.2

A battery tester is used to online test the remaining capacity of the battery.

#### 4.2.3. Process and Analysis of Comparative Experiments

In the first experiment, all sensor probes were fixed in one container that is full of water from group 3. The depth of the sensor probes during this trial was fixed at approximately 0.3 m beneath the water surface, which was similar to the deployment position in real lake water. To validate the proposed approach, different decision approaches were applied on the three monitoring nodes mentioned. Node 1 works continuously with a constant sampling interval of 30 s and communicating interval of 5 min. Node 2 uses a basic method to make the communication device sleep after a certain period to approximately suit the electric quantity of the battery. It has a constant sampling interval and communicating interval similar to that of Node 1. However, the communicating device enters a dormancy state for 0.5 h after working continually for 2 h. Node 3 adopts the approach proposed in this paper. It self-configures the sampling interval and communicating interval or enters a dormancy state with the decision of the reasoning engine which considers four rules mentioned in Section 3.3. The monitored water sample is the same as that of the three nodes. The comparison of the three nodes is shown in Table 3.

**Table 3:** Comparison of the three nodes.

Node Name	Control Rules	Description	Hardware
Node 1	Works continually without any control.	Has a constant sampling interval of 30 s and communicating interval of 5 min.	All nodes have hardware similar to those mentioned.
Node 2	Makes the communication device sleep after a certain period to approximately suit the electric quantity of the battery.	Has a constant sampling interval and communicating interval same as those of Node 1. However, the communicating device enters a dormancy state for 0.5 h after working continually for 2 h.	
Node 3	Uses the approach proposed in this paper.	Self-configures the sampling interval and communicating interval or enters a dormancy state with the decision of the reasoning engine, which considers four rules mentioned in Section 3.3.	

The same types of battery as those mentioned were used to provide energy for three nodes. Thus, consumed energy is the total energy used by the monitoring nodes to perform sensing, transmission, and reception. Every battery was fully charged. Then, the monitoring nodes began to perform data collection and communication. Each of the four parameters (water temperature, pH, DO, and conductivity) were collected and transmitted. The dump energy of three battery groups were tested every 30 min until the battery power ran out. The test result was the average of three experiments.

**Figure 19:**
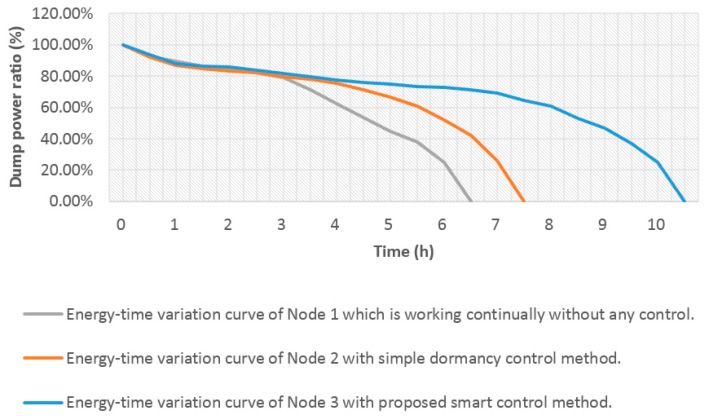
Test results of durations of three nodes for the same battery consumption with different control method.

Figure 19 shows the dump power ratio of three monitoring nodes for the same battery consumption. Electric quantity (vertical) decreased with time (horizontal). Among the three nodes, Node 1 had the shortest duration. Node 2 had a longer duration than Node 1 for the same energy consumption. Node 3 conserved the most energy. These results show that because the communication device of Node 2 entered a dormancy state at a certain moment, it sustained a slightly longer duration than Node 1. For Node 3, the ontology middleware played a role in saving energy by deciding to reconfigure the monitoring node according to the energy rule. Therefore, Node 3 had the longest duration. The ontology middleware did not affect the accuracy of the water-quality data, but it effectively reduced the energy consumption of the WSN.

In the next experiment, two monitoring nodes were adopted. Each node had the same hardware as that mentioned in Section 4.2.1. Each monitoring node was connected with four sensors (water temperature, pH, DO, and conductivity) as mentioned in Section 4.2.2. All nodes adopted the same approach proposed in this paper and the same as that of Node 3 in Table 3. However, the monitored water samples are different. To verify the energy saving ability with the changing of water quality, five groups of water sample were used, which corresponded to the five grades of water quality as shown in Table 2. Each group was placed in a separate container. The sensor probes of Node 1 were fixed in the container, which contained the worse quality of water group 5. The sensor probes of Node 2 switching location from one container to the others lasted for two hours each. The switching sequence is as follows: Water group 5—Water group 4—Water group 2—Water group 1—Water group 3. Table 2 shows that except the water temperature, all the parameters are different in the five water groups, especially the DO and conductivity. Therefore, in our simulation, Node 1 was working in a steady water environment, meanwhile Node 2 was working in a water environment with changing water parameters. However, the water parameters changed in our simulation. Every battery was fully recharged again, and the monitoring nodes began performing data collection and communication. The dump energy of the three battery groups was tested.

**Figure 20:**
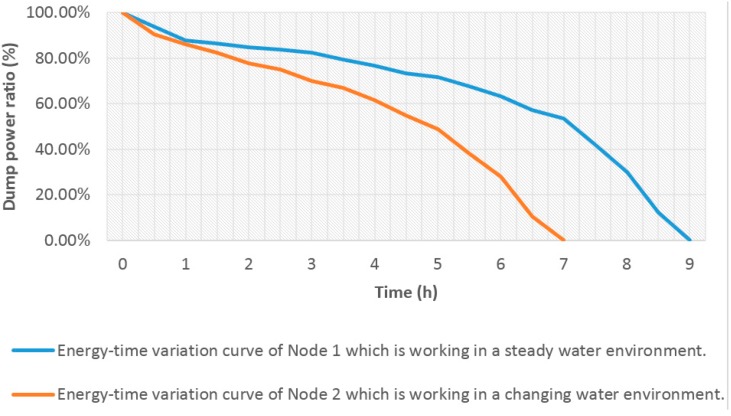
Test results of durations of two nodes for the same battery consumption with different monitored water samples.

Figure 20 shows the test result of the average of three experiments. Node 1 had a longer duration than Node 2 for the same energy consumption. This result means that the ontology middleware reconfigured the WSN at different grades of water sample and effectively reduced the energy consumption. The ontology middleware played a role in saving energy, which decided to reconfigure the monitoring node according to the data rule.

**Figure 21:**
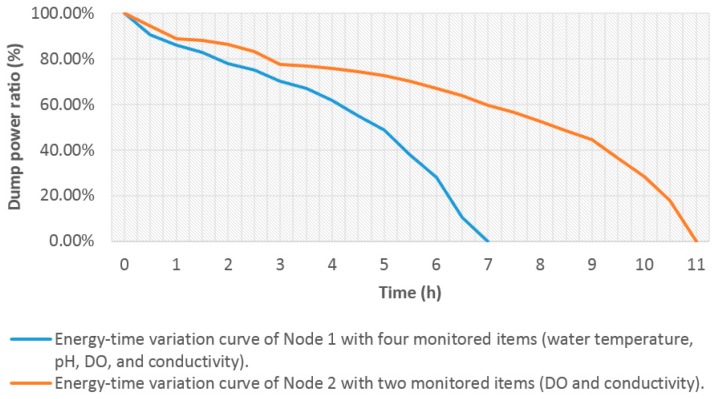
Test results of durations of two nodes for the same battery consumption with different monitored items.

Finally, two monitoring nodes that had the same hardware as mentioned in Section 4.2.1 were adopted. Each monitoring node was still connected with the four sensors (water temperature, pH, DO, and conductivity) as mentioned in Section 4.2.2. All the sensor probes of the two monitoring nodes were fixed in the container of Water group 5. The same approach proposed in this paper as for Node 3 in Table 3 was adopted for two monitoring nodes. We set the observed objects of water temperature and pH to unobserved in the management interface with Node 2. Node 1 still monitored four items (water temperature, pH, DO, and conductivity). Every battery was fully recharged again, and the monitoring nodes began performing data collection and communication. The dump energy of the three battery groups was tested. Figure 21 shows the test result of the average of the three experiments. 

Node 2 had a longer duration than Node 1 for the same energy consumption. This result means that the ontology middleware that makes the water temperature sensor sleep and the pH sensor of Node 2 have an effect on energy consumption. The observation demand state rules play a role in decision making.

## 5. Conclusions

The application of an ontology results in a clear structure. It uses a natural assumption that is highly suitable for pervasive computing systems. The accurate expression of knowledge is essential when automatically monitoring, identifying, and processing a WSN working state. SWRL is used to describe the reasoning rules for an OWL instance and to infer new knowledge. By using SWRL, we have improved the ontological reasoning functions of the WSN.

This paper proposes a method using a middleware based on ontology and SWRL rules for WSN state monitoring and operational decision support. We considered a WSN for lake water quality monitoring. The structure and state information is encoded in the WSN ontology, and can be used for real-time monitoring of the device, data, object observation, and water quality states. A rule set based on SWRL combined with the ontology can help us make intelligent decisions. Our case study shows that the proposed method effectively reduced the energy consumption and improved the reliability of the WSN.

In the future, we will improve the monitoring functions of the WSN ontology. We will also add more devices, and include more complex strategies in the reasoning rules that dynamically control the energy consumption; for example, topology protocols, power aware routing protocols, and more effective sleeping management protocols. We will conduct more experiments at a larger scale to test rule conflicts and the diagnosis accuracy.
